# Encephalocraniocutaneous Lipomatosis, a Radiological Challenge: Two Atypical Case Reports and Literature Review

**DOI:** 10.3390/brainsci12121641

**Published:** 2022-11-30

**Authors:** Magdalena Machnikowska-Sokołowska, Piotr Fabrowicz, Jacek Pilch, Weronika Roesler, Mikołaj Kuźniak, Katarzyna Gruszczyńska, Justyna Paprocka

**Affiliations:** 1 Division of Diagnostic Imaging, Department of Radiology and Nuclear Medicine, Faculty of Medical Sciences in Katowice, Medical University of Silesia, 40-752 Katowice, Poland; 2 Diagnostic Imaging and Interventional Radiology Centre, The Independent Public Clinical Hospital No. 6, Medical University of Silesia, 40-752 Katowice, Poland; 3 Department of Pediatric Neurology, Faculty of Medical Sciences in Katowice, Medical University of Silesia in Katowice, 40-752 Katowice, Poland; 4 Student’s Scientific Society, Department of Pediatric Neurology, Faculty of Medical Sciences in Katowice, Medical University of Silesia in Katowice, 40-752 Katowice, Poland

**Keywords:** encephalocraniocutaneous lipomatosis, ECCL, Haberland syndrome, neuroimaging, child brain, MRI, CT, brain calcifications

## Abstract

Encephalocraniocutaneous lipomatosis (ECCL; Haberland syndrome, #613001) is an extremely rare congenital disorder that is manifested by the involvement of the skin, eyes and central nervous system (CNS). We report two cases of children with ECCL diagnosis. First was an 8-year-old girl who presented with symptomatic epilepsy, cerebral palsy and developmental delay. In 2020, she was admitted to the hospital due to the exacerbation of paresis and intensified prolonged epileptic seizures, provoked by infection of the middle ear. Diagnostic imaging revealed radiological changes suggestive of ECCL, providing a reason for the diagnosis, despite the lack of skin and eye anomalies. The second child, a 14-year-old girl, was consulted for subtle clinical signs and epilepsy suspicion. Diagnostic imaging findings were similar, though less pronounced. Based on neuroradiological abnormalities typical for Haberland syndrome, the authors discuss possible ECCL diagnosis.

## 1. Introduction

Encephalocraniocutaneous lipomatosis (ECCL, #613001) also known as Haberland or Fishman syndrome [1], is an extremely rare congenital neurocutaneous disorder presenting usually with unilateral craniofacial or neck lipomas, as well as unilateral eye and brain tissue lesions [2,3,4]. Typically, there are central nervous system (CNS), periorbital and/or skin manifestations, consisting of brain anomalies such as unilateral hemispheric atrophy, intracranial calcifications, meningeal lipomatosis, arachnoid cysts, ventriculomegaly, ipsilateral lipomas or lipodermoids of the head and neck, including the periorbital region. Morphological changes are often accompanied by epileptic seizures and hemiplegia, as well as psychomotor and intellectual developmental delay [5]. The clinical spectrum is broad and highly variable, from mild forms of oculoectodermal syndrome (OES) with eye and skin abnormalities in most cases, to severe encephalocraniocutaneous lipomatosis [6].

Imaging of Haberland syndrome consists of head computed tomography (CT) for calcification assessment and magnetic resonance imaging (MRI) showing a rather typical imaging pattern, as above. In some cases with OES, arachnoid cyst is the only abnormality reported in neuroimaging. Previously, ECCL was thought to be a one-sided disease, but according to Moog, 40 % of patients have bilateral skin/eye changes [6]. The first reports of this syndrome date back to 1970 by Catherine Haberland and Maurice Perou [4], and to date, ca. 85 cases have been described in the literature [6]. The authors report two cases with neuroradiological abnormalities typical for Haberland syndrome without skin/eye/other organ abnormalities and discuss possible ECCL diagnosis.

## 2. Case Reports with Clinical Findings

### 2.1. Case 1

The girl was born at 37 weeks of gestation through vaginal delivery. The dilatation of the left lateral ventricle with a hyperechoic choroidal plexus was found in the postnatal transcranial ultrasound. The patient presented signs of general developmental delay. From the age of two, the patient presented with epileptic seizures. Episodes were initially febrile, which later developed into fever-independent seizures with an average frequency of 1–2 times per month. At the age of 3, she was diagnosed with cerebral palsy, characterised by the right-sided paresis; and symptomatic epilepsy, which was treated with valproic acid and carbamazepine.

At the age of 8, the girl was admitted to the Pediatric Neurology Department due to right-sided pyramidal severe paresis and intensified prolonged epileptic seizures.

She was diagnosed with concomitant otitis media, which could be the cause of the symptoms’ exacerbation. After successful treatment of the otitis media and further administering of antiepileptic drugs, the child’s condition improved significantly.

Head CT was performed to exclude acute, neurosurgery-requiring conditions. Surprisingly, radiological analysis indicated Haberland syndrome as a potential diagnosis. 

### 2.2. Case 2

A 14-year-old girl was referred to a genetic outpatient clinic for further MRI diagnosis of head abnormalities and suspicion of epilepsy. Pregnancy and delivery were uncomplicated; family history was negative. Psychomotor development was normal. 

Formerly, she had remained under neurological observation with suspicion of epilepsy. At the age of 10, there was an episode of facial grimace on the right side and loss of sensation in the right hand area, without disturbance of consciousness. There were also a few short sensory disturbances in the right hand without disturbance of consciousness. A report of the MRI of the head at that stage stated the presence of meningeal angiomatosis. Physical examination showed no skin and ophthalmic changes typical of Sturge–Weber syndrome, but subtle facial asymmetry was visible, with slight hypotrophy on the right. Discreet asymmetry of the face when smiling was observed. Neurological examination was normal. Electroencephalography (EEG) recording revealed no epileptic discharges and antiepileptic treatment was not indicated. The girl did not meet the criteria for the clinical diagnosis of Sturge–Weber syndrome, and Haberland syndrome was suspected. 

## 3. Radiological Findings

### 3.1. Methods 

Head CT was performed with a 16-row SIEMENS Somatom Definition AS scanner, using spiral technique and with the diagnostic protocols adjusted to child age, slice thickness 0.5 mm and 100 KV. The obtained CT axial images were analysed using a dedicated workstation—Syngo.via 202—Siemens Healthineers. 

Brain MRI was performed using Artist 1.5 T GEM (General Electric, GE Healthcare, Milwaukee, WI, USA). Imaging protocol included standard sequences: T1, T2, T2 Flair, DWI (diffusion-weighted imaging) and SWI (susceptibility-weighted image) and T1 3D postcontrast. The dose of gadolinium contrast was 0.1 mL/kg of body weight. The data were transferred to a commercially available Workstation ADW 3.2.

### 3.2. CT/MRI Abnormalities

**Figure 1:**
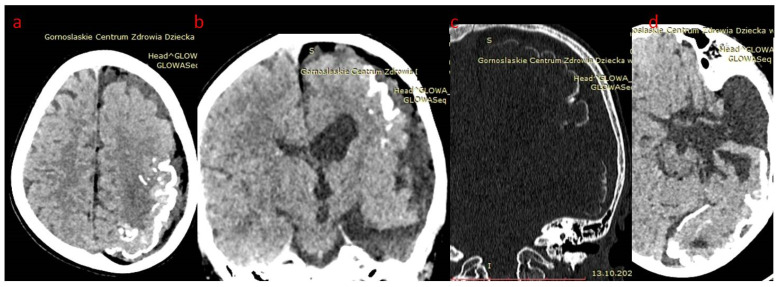
(**a**–**d**) Patient 1. Head CT brain window: (**a**) axial and (**b**) reformatted coronal plane; hemiatrophy of left hemisphere, left ventriculomegaly, meningeal lipomatosis, arachnoid cyst in temporal fossa and gyral calcifications; (**c**) bone window coronal reformatted plane: extensive left sided gyral calcifications; (**d**) axial plain—left arachnoid cyst.

**Figure 2:**
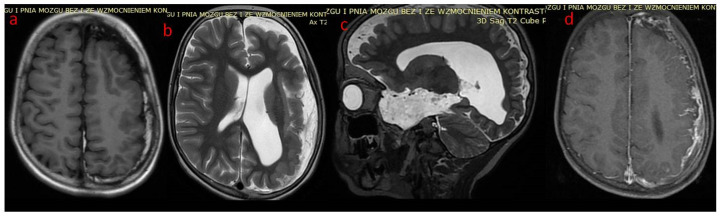
(**a**–**d**) Patient 1. Brain MR (**a**) axial T1—left-sided polymicrogyria, hemiatrophy and meningeal lipomatosis; (**b**) axial T2—left-sided hemiatrophy, ventriculomegaly and polymicrogyria; (**c**) sagittal T2—as above, and left temporal space enlargement (arachnoid cyst) with lipomatous tissue; (**d**) axial T1 with contrast—meningeal enhancement along gyri of left hemisphere.

**Figure 3:**
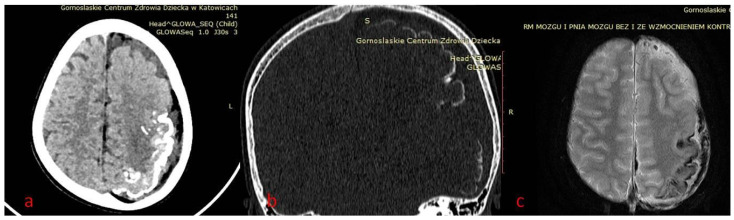
(**a**–**c**) Patient 1. Brain CT—left parietal and temporal gyral calcifications. (**a**,**b**) CT in axial brain window and coronal reformatted bone window; (**c**) MRI SWI sensitive to calcium/blood metabolites.

In case 1 (Figure 1, Figure 2 and Figure 3), cranial CT examinations revealed a significant reduction in the left brain hemisphere in comparison to the right, with the presence of streaked calcifications in the parieto-occipital cortex and a large congenital arachnoid cyst of the left temporal pole, ventriculomegaly. Fat tissue within meninges surrounding the left hemisphere was also present. Subsequently, magnetic resonance imaging was performed, confirming findings above in the form of lipomatosis around the left brain hemisphere, calcifications, arachnoid cysts and left side meningeal angioma, and additionally revealing areas of polymicrogyria and meningeal postcontrast enhancement of the left hemisphere. 

**Figure 4:**
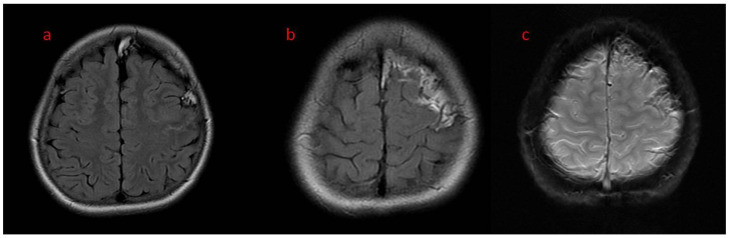
(**a**–**c**) Patient 2. Brain MR in axial plane. (**a**,**b**) T2 flair—left-sided frontal meningeal lipomatosis; (**c**) SWI—subtle calcification artifacts.

**Figure 5:**
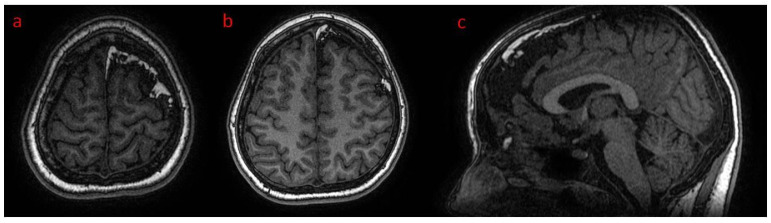
(**a**–**c**) Patient 2. Brain MR (**a**,**b**) axial T1- left-sided meningeal lipomatosis; (**c**) sagittal T1—minimal space enlargement without arachnoid cyst with lipomatous tissue.

**Figure 6:**
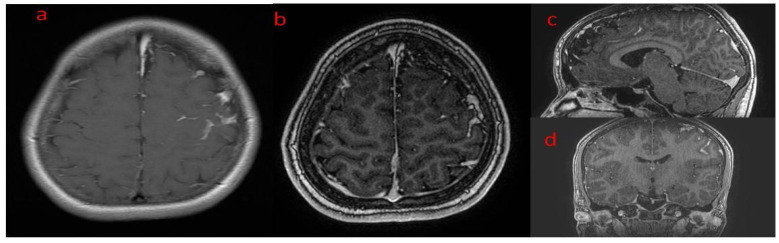
(**a**–**d**) Patient 2. Brain MR, T1 sequence after contrast enhancement: (**a**) axial; (**b**) thin-slice axial; (**c**,**d**) T1 multiplanar reconstructions: (**c**) sagittal and (**d**) coronal–meningeal enhancement along gyri of left hemisphere—frontal lobe.

**Figure 7:**
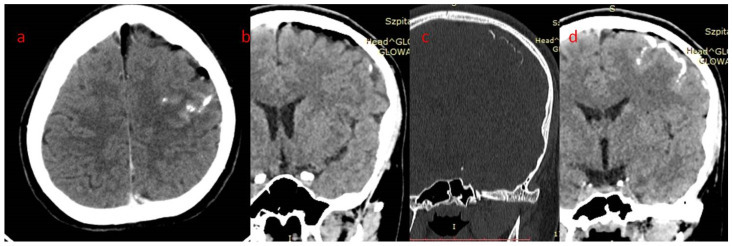
(**a**–**d**) Patient 2. Brain CT: gyral calcifications in left frontal lobe, coexisting subtle arachnoid space enlargement with small fat tissue amount. (**a**,**b**) Axial and reformatted coronal brain window—fat tissue; (**c**,**d**) left frontal calcifications: coronal reformatted (**c**) bone window and (**d**) brain window.

In case 2 (Figure 4, Figure 5, Figure 6 and Figure 7), enlarged asymmetrical, left-sided frontal arachnoid spaces were filled with lipomatous tissue; calcifications along pia matter were seen with subtle contrast enhancement. No arachnoid cyst appeared up to this point, and no migration anomalies were described. CT of the head was shown to be similar to case 1, but with less-pronounced radiological changes. 

In the case of our patients, diagnostic imaging proved to be crucial and significantly promoted the diagnosis of ECCL.

## 4. Discussion

### 4.1. Aetiology 

The aetiology of the OES/ECCL spectrum is related to the low percentage of mosaicism of activating pathogenic variants identified in *FGFR1* (8p11.23, #136350) and *KRAS* (12p12.1, *190070) genes in DNA derived only from affected tissues [7]. Evaluation of DNA from blood, buccal swabs or saliva did not reveal mosaic pathogenic variants. Vasculogenesis dysfunctions and constitutive activation of RAS-MAPK may also influence the pathogenesis of Haberland syndrome [8,9]. Mosaic pathogenic variants in the *NRAS* gene should be taken into consideration, especially in patients with OES phenotypes [10,11].

### 4.2. Clinical Presentation and Diagnostic Findings with Literature Reference

Clinical variability is probably related to involved tissue, type of pathogenic variants and percentage of mosaicism. Developmental abnormalities most often involve the CNS, eyes and scalp, but may also be accompanied by defects of other internal organs [2,12].

Clinical presentation of Haberland syndrome may be varied, but a specific set of features is considered characteristic of the disorder. The patient may have spasticity, facial palsy, hemiparesis or hemiplegia [13]. Psychomotor development is usually delayed with associated drug-resistant epilepsy, but in rare cases the course of the disease can be mild, with normal psychomotor development. The absence of seizures and normal mental development has only been reported in a few cases [12]. Our older patient presented with such a normal development that is atypical for ECCL, and antiepileptic treatment is not necessary at the moment.

In imaging examination, lipomas of the central nervous system are the hallmark lesions of encephalocraniocutaneous lipomatosis [12], but it is possible to find other CNS anomalies, including asymmetric cerebral atrophy, leptomeningeal angiomatosis, arachnoid cysts, widening of the subarachnoid spaces, dysplastic cortex, agenesis of the corpus callosum, calcifications, porencephalic cysts or dilated ventricles [9]. In some cases, cardiac lipomatosis was also described.

Imaging of ECCL should comprise both CT and MRI. Calcifications would be best seen in CT of the head, and due to ionising radiation, this method should be used with caution just for full detection of calcium deposits. All other features of disease are best visualised in MRI, and this is a method of choice for paediatric brain imaging. MRI will also be irreplaceable for the follow-up, which must be included in the algorithm because of possible neoplasmatic transformation. An increase in intracranial pressure due to cyst or ventricle enlargement may need neurosurgical treatment; therefore, follow-up imaging of the brain to prevent acute hydrocephalus is also necessary.

In addition, Haberland syndrome is manifested by eye anomalies (mostly corneal dermoids or epibulbar dermoids, calcification of globe) and skin lesions (non-scarring alopecia, naevus psiloliparus, subcutaneous fatty deposits and skin tags), which are usually unilateral, but occasionally they could also be bilateral, and typically occur in distinct patterns [2,14,15].

Clinical diagnosis of Haberland syndrome is based on Moog criteria, while neuroradiologists can raise suspicions of the ECCL spectrum based on the coexistence of typical intracranial pathologies [16]. Intracranial lipomatosis, arachnoid cysts, cerebral calcifications and hemispheric asymmetry in a patient, especially with skin and eye lesions, can lead to proper diagnosis. 

In 2006, Hunter proposed the initial diagnostic criteria for ECCL. In 2009, Moog revised the criteria for definite/proven and possible ECCL cases; the third group of probable ECCL cases was excluded [2,3]. The newest comprehensive summary and review of clinical and genetic data with clinical diagnostic major and minor criteria was presented by Moog and Dobyns in a 2022 article [6]. One major and three minor criteria are included in the revised ECCL criteria regarding ocular system involvement; one major and five minor criteria are included regarding cutaneous system involvement; and three major and six minor criteria are included regarding CNS involvement. Three major criteria are also seen involving the other systems [2]. Table 1 summarises the above criteria. Applications of these criteria are described in Table 2. 

In the presented patients, there are no skin or ophthalmic lesions typical for ECCL, which would allow for a certain clinical diagnosis. However, there are changes within the CNS that raise the suspicion of Haberland syndrome. It is known that the ECCL exhibits high phenotype variability due to the presence of mosaic pathogenic variants. The presence of pathogenic variants only within the CNS cannot be ruled out. Genetic confirmation is possible only by performing DNA tests isolated from diseased tissues. The lack of skin and ophthalmic changes does not allow for a biopsy to perform genetic tests, and at the same time, there are no clinical indications and there is no parental consent for a brain biopsy. On the other hand, brain imaging of our patients shows radiological CNS abnormalities that are typical for ECCL, not corresponding to any other discussed diseases, which we suggest would still make Haberland disease diagnosis at least probable.

### 4.3. Differential Diagnosis

The following conditions may be taken into consideration in differential diagnosis of ECCL:

#### 4.3.1. Proteus Syndrome (14q32.33, #176920)

Proteus syndrome (PS), similarly to ECCL, is classified as an extra-rare disease in which patients may present with lipomas, among other symptoms. However, in contrast to ECCL, PS is an progressive disease characterised by asymmetric, disproportionate overgrowth, development of linear epidermal naevus or specific tumours, including bilateral ovarian cystadenoma and parotid monomorphic adenoma [18].

#### 4.3.2. Oculocerebrocutaneous Syndrome (OCCS; Delleman–Oorthuys Syndrome, #164180)

OCCS is a rare disorder characterised by skin, eye and brain anomalies. 

Its most common skin features are unusual skin appendages (pedunculated, moveable, finger-like) as well as focal hypoplastic or aplastic skin defects that are also found in ECCL. However, the most characteristic skin anomalies for ECCL are naevus psiloliparus and subcutaneous lipomas, which do not develop in OCCS. 

Brain anomalies found in OCCS are unique and recognisable. Complex malformations of cortical development with cortical and subcortical components vary significantly from brain anomalies in ECCL (intracranial lipomas, abnormal intracranial vessels, arachnoid/cysts or calcifications).

Typical eye defects in OCCS are congenital orbital cysts with cystic microphthalmia, and these aberrations are not characteristic for ECCL. In the latter case, the typical features are chormistomas, colobomas and calcifications of the globe [19]. Currently, oculocerebrocutaneous syndrome is thought to be on the mild spectrum of ECCL changes [20].

#### 4.3.3. Goltz Syndrome (Gorlin–Goltz Syndrome, 1p34.1, 9q22.32,10q24.32 #109400)

This is a rare genetic condition with an autosomal-dominant pattern of inheritance. CNS calcifications, eyelid microcysts and jaw tumours occur in both Goltz Syndrome and Haberland Syndrome and may cause misdiagnosis. Nevertheless, the presence of other characteristics for Goltz Syndrome aberrations, such as basal cell carcinoma, millia, palmar and plantar pits, hypertelorism or skeletal and urogenital anomalies, may help in the diagnostic process [21]. 

#### 4.3.4. Sturge–Weber Syndrome (SWS, 9q21.2, #185300)

In this rare condition, the following anomalies can usually be observed: hemiatrophy and gyriform calcifications of the brain and abnormal intracranial vessels. However, intracranial lipomas are not present in this syndrome, and the age of calcifications appearing differs: in ECCL they may be found earlier, whereas in SWS most often not before the first year of life. In contrast to ECCL, characteristic choristomas in glaucomas commonly occur in SWS [22].

### 4.4. Treatment

Currently, there is no specific treatment for patients with Haberland syndrome. Standard management is symptomatic. It includes surgical correction of ocular and cutaneous lesions and anticonvulsant therapy for seizures. A regular follow-up of psychomotor development, continuation of rehabilitation and development stimulation is also recommended. Cancer screening in patients with this condition is indicated [23], due to higher risk of brain tumours associated with ECCL, such as low-grade glioma [9], pilocytic astrocytoma [24] and possibly Wilms tumour [25]. In our patients, no tumour diagnosis has been made so far.

## 5. Conclusions

The poor presence of noncerebral symptoms makes our cases distinctive from others and leads to the possibility of more frequent occurrence of ECCL.

We propose to consider the possibility of extending the clinical phenotype of ECCL limited to isolated changes in the CNS, revalidating criteria for diagnosing Haberland syndrome. Further analyses and possible genetic tests of a greater number of similar patients with lesions limited to the CNS are necessary for higher detectability. This would make a great impact, because misdiagnosed patients can be mistreated, which in turn can lower the quality of their life as well as of their caregivers.

## Figures and Tables

**Table 1:** The diagnostic criteria proposed by Hunter for the ECCL and modified by Moog [2,17].

System	Major Criteria	Minor Criteria
Skin	Proven naevus psiloliparus (NP) Possible NP and >1 of the minor criteria 2–5 >2 minor criteria 2–5	Possible NP Patchy or streaky non-scarring alopecia without fatty naevus Subcutaneous lipoma(s) in frontotemporal region Focal skin aplasia/hypoplasia on scalp Small nodular skin tags on eyelids or between outer canthus and tragus
Ocular	Choristoma with or without associated anomalies	Corneal and other anterior chamber anomalies Ocular and eyelid coloboma Calcification of globe
Central Nervous System	Intracranial lipoma Intraspinal lipoma Two of the minor criteria	Abnormal intracranial vessels, e.g., angioma, excessive vessels Arachnoid cysts or other abnormalities of meninges Complete or partial atrophy of hemisphere Porencephalic cyst(s) Asymmetrically dilated ventricles or hydrocephalus Calcification (not basal ganglia)
Other Systems	Jaw tumour (osteoma, odontoma, ossifying fibroma) Multiple bone cysts Coarctation of aorta	

**Table 2:** ECCL application criteria for diagnosis [2].

Definite Case	Probable Case
Three systems involved, major criteria >2, OR Three systems involved, proven naevus psiloliparus (NP) NP OR possible NP + >1 minor skin criteria 2–5 Two systems involved with major criteria, one of which is proven NP OR possible NP + >1 of minor skin criteria 2–5	Two systems involved, major criteria in both Two systems involved, proven or possible NP

## Data Availability

Not applicable.

## Abbreviations

ECCL	(Encephalocraniocutaneous lipomatosis—Haberland syndrome)
CNS	(Central nervous system)
OES	(Oculoectodermal syndrome)
CT	(Computed tomography)
MRI	(Magnetic resonance imaging)
EEG	(Electroencephalography)
DWI	(Diffusion-weighted imaging)
SWI	(Susceptibility-weighted image)
NP	(Naevus psiloliparus)
PS	(Proteus syndrome)
OCCS	(Oculocerebrocutaneous syndrome (OCCS; Delleman–Oorthuys syndrome)
SWS	(Sturge–Weber syndrome)
